# A framework to quantify flow through coral reefs of varying coral cover and morphology

**DOI:** 10.1371/journal.pone.0279623

**Published:** 2023-01-18

**Authors:** Andrew W. M. Pomeroy, Marco Ghisalberti, Michael Peterson, Vahid Etminan Farooji

**Affiliations:** 1 Oceans Graduate School, The University of Western Australia, Crawley, Western Australia, Australia; 2 The UWA Oceans Institute, The University of Western Australia, Crawley, Western Australia, Australia; 3 School of Engineering, The University of Western Australia, Crawley, Western Australia, Australia; University of Guam, GUAM

## Abstract

Flow velocities within coral reefs are greatly reduced relative to those at the water surface. The in-reef flow controls key processes that flush heat, cycle nutrients and transport sediment from the reef to adjacent beaches, all key considerations in assessments of reef resilience and restoration interventions. An analytical framework is proposed and tested with a suite of high-resolution numerical experiments. We demonstrate a single parameter that describes the total coral frontal area explains variation of horizontally averaged velocity within a reef canopy across morphologies, densities, and flow depths. With the integration of existing data of coral cover and geometry, this framework is a practical step towards the prediction of near-bed flows in diverse reef environments.

## Introduction

The hard coral colonies that form coral reef benthos are irregular in form and vary on spatial scales that range from centimeters to kilometers. This irregular and complex form directly modifies the in-reef flow that occurs between the seabed and the top of the coral colonies [1, 2]. While this in-reef flow is often well mixed, it can control biological processes such as the capture of prey by corals [3], the rate of nutrient uptake [4] and coral respiration and calcification [5]. It also governs a range of physical processes that are responsible for the attenuation of waves [6–8] and currents [9] as well as the transport of reef-generated sediment [1, 10]. These physical processes ultimately shape the adjacent shoreline and reduce the risk of coastal flooding and erosion [11], which motivates many environmental restoration activities [12]. Given the controlling influence of the flow within the reef benthos [13] as well as the role of benthos on energy dissipation by drag in particular [9], there is a clear need for a practical framework that allows the prediction of in-reef flow across a wide range of colony types, coral cover and flow speeds.

**Fig 1:**
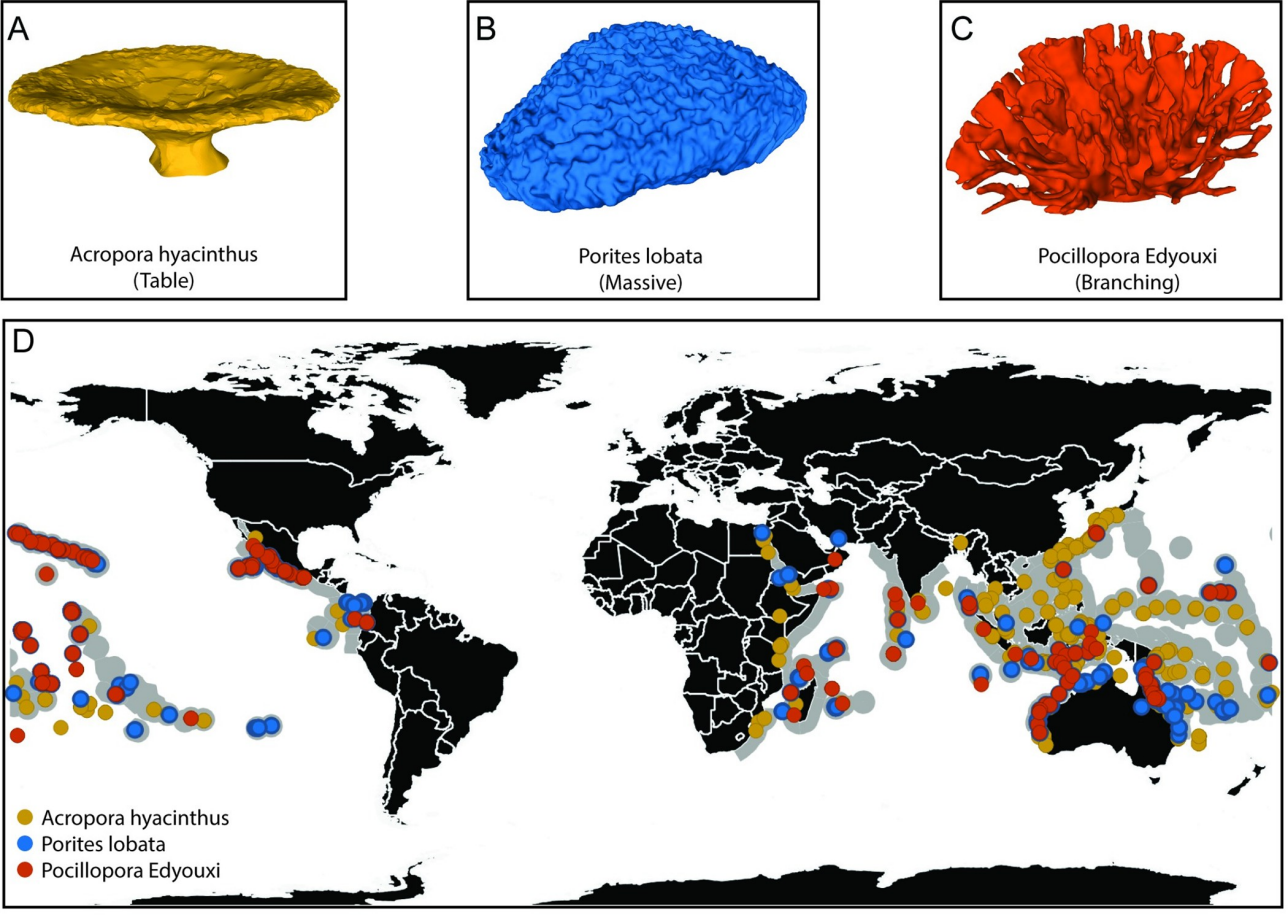
The global distribution of the three archetypal benthos colony forms analyzed here (“table”, “massive” and “branching”). Recorded observations [18] of the representative species (A) *Acropora hyacinthus* [15], (B) *Porites lobata* [15] and (C) *Pocillopora edyouxi* [16] are indicated by colored dots in (D) with grey shading indicating ecoregions [19] where these broad morphology types are likely to be found [17]. Panels A-C compare example morphologies of each representative species with the model forms embedded within numerical and experimental simulations (derived from reef colonies digitized in the field, kindly provided by The Hydrous, http://www.thehydro.us). The three coral archetypes are broadly representative of a reef benthos observed globally.

Despite the wide range of coral species globally, coral colonies can be broadly classified from a physical perspective based on their morphological form [14]. For example “plate” or “table” morphologies such as *Acropora hyacinthus* [15] are characterized by large flat surfaces (Fig 1A), “massive” morphologies such as *Porites lobata* [15] form monolithic structures (Fig 1B), and “branching” morphologies such as *Pocillopora edyouxi* [16] create a complex porous array (Fig 1C). A wide range of coral colonies globally are morphologically similar to these three archetypes [17] and indeed these three species alone have been observed [18] throughout many of the eco-regions where coral reefs are located (Fig 1D).

Many reef systems are current-dominated systems, such as those located in macro-tidal regions [20] and reefs located in the relatively deep lagoons of barrier reefs [21] and atolls [22, 23]. For the more than two-thirds of reefs that experience wave-driven flows [24], there are energetic wave-breaking zones on the forereef slope [2]. This can generate significant cross-reef currents [25–27] and small depth-limited waves [8], such that the steady component of the flow (and its attenuation by the reef) is still significant [28, 29]. In steady flows, the drag exerted by the coral colonies creates a substantial reduction in the spatially-averaged in-reef flow, relative to the near-surface flow [28, 30, 31]. In addition to this vertical variation, the generation of low-velocity wake regions behind coral colonies (and the individual ‘branches’ of some species), means that in-reef flows exhibit significant horizontal variability [32]. Similar processes of attenuation are also observed in oscillatory flows, except that this attenuation is typically much less than that observed in steady flows [33]. Thus, quantification of steady flow attenuation is of particular importance, has broad applicability and can serve as a foundation to help explain the observed variation of in-reef flow with coral morphology and cover under idealized conditions.

In this study, we present a practical framework for predicting the ratio of horizontally-averaged in-reef and near-surface velocities for the three coral archetypes in Fig 1. Our numerical approach considers steady currents over a single coral colony, with one of three morphologies that broadly represent the global range, within a domain with cyclic boundaries (thus representative of an infinite reef). We first present the theoretical basis for the framework. We then explain how the framework was validated using high resolution Computational Fluid Dynamics (CFD) numerical models. We also describe the parameter domain that we explore numerically to test the broad applicability of our framework as well as validate the numerical model in the laboratory. Next we present the results from the validation and numerical simulations and demonstrate the first-order accuracy of the predictive framework. Finally we discuss the performance of the framework, how this framework can be extrapolated to mixed reef environments, as well as how the application of this framework may assist in understanding a wide range of reef processes.

## Methods

### Analytical framework for in-reef velocity

**Fig 2:**
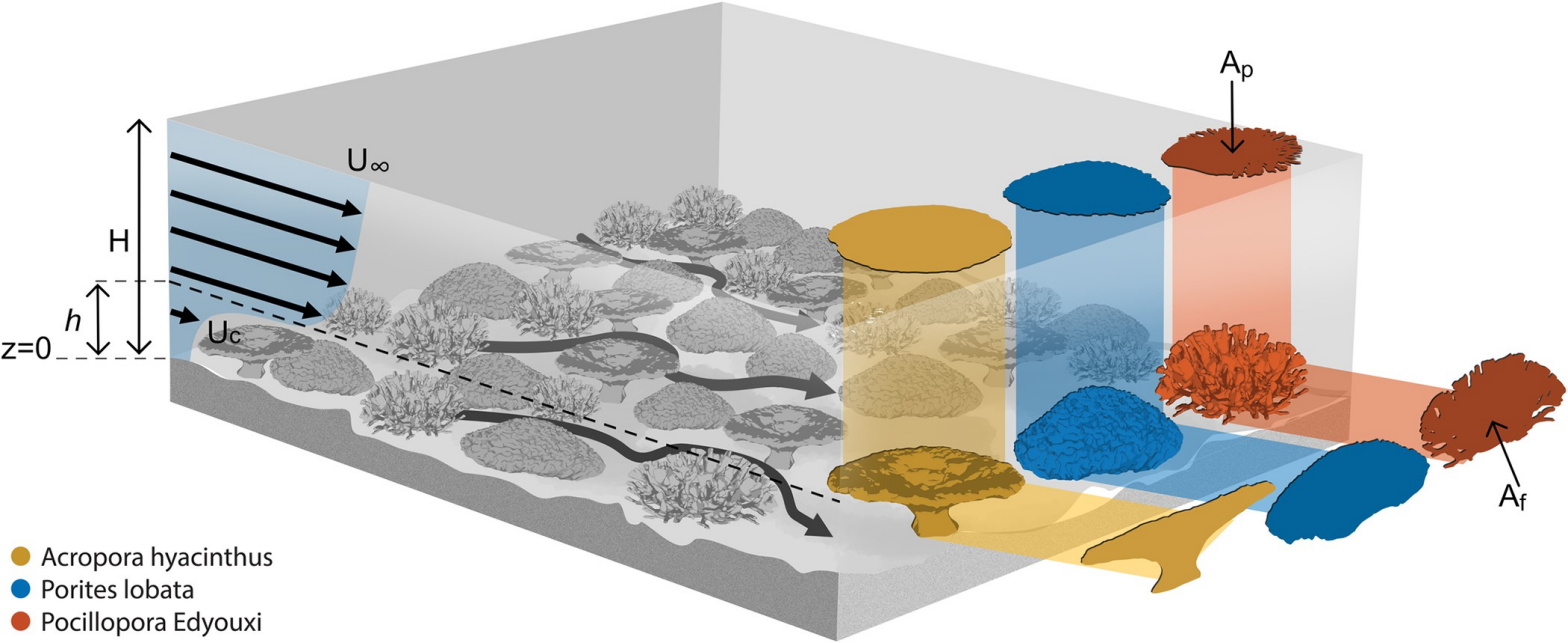
Conceptual model of the flow above and within benthos on a coral reef. For a given water depth (*H*), the near-surface flow velocity (*U*_*∞*_) is greater than that within the reef region (*U*_*c*_), which is defined by the height of the benthos (*h*) above the seabed. The plan area (*A*_*p*_) and frontal area (*A*_*f*_) are shown for the three archetypal coral forms in Fig 1 (“Table”, “Massive” and “Branching”). Note that *Af* is not simply the total coral area projected into the flow; rather, it is the sum of the projected areas of all coral surfaces.

The analytical framework presented here builds on previous work by others, such as that by Lowe et al [33]. It predicts the ratio (*β*) of the horizontally- and depth-averaged in-reef velocity (*U*_*c*_, Fig 2) to the velocity well above the reef benthos (*U*_*∞*_):

$$\beta =\frac{U_{\infty }}{U_{c}}\equiv 1+\frac{\Delta U}{U_{c}},$$ (1)

where *ΔU* is the difference between *U*_*∞*_ and *U*_*c*_.

The attenuation of the in-reef flow occurs due to the substantial drag forces imposed by coral reef colonies. For a given coral colony, this drag is typically described through a quadratic law relating the drag force (*F*_*D*_) to the depth‐averaged in-reef flow velocity (*U*_*c*_) and the area of the coral colony projected into the flow (the “frontal area” in Fig 2, *A*_*f*_):

$$F_{D}=\frac{1}{2\phi }\rho C_{D}A_{f}U_{c}^{2},$$ (2)

where *ρ* is the density of water, *C*_*D*_ is a drag coefficient and *ϕ* is the porosity of the reef colony, which was calculated by treating the canopy as a box with the upper limit defined at the top of the coral morphology (*z* = *h*); the porosity is then equal to one minus the ratio of coral solid volume to box volume. We discuss the limitations of this approach to some morphologies such as plate corals later in the discussion. While complex forms such as branched corals (e.g., *Pocillopora edyouxi*) have been suggested to act as bluff bodies [34], *A*_*f*_ is not simply the total coral area projected into the flow; rather, it is the sum of the projected areas of all coral surfaces (as all coral surfaces will exert drag and create flow attenuation). What this means is that, for example, for a branching coral each branch along the flow direction axis contributes to increase *A*_*f*_. We note this is not trivial for practical applications and we propose an approach to address this later. In this study, we evaluate the area projected into the incoming flow of each individual element (e.g., each individual branch) of each morphology and define *A*_*f*_ as the sum of these projected areas.

When this is extrapolated to the reef scale, the drag force exerted per unit reef area and unit fluid density is:

$$f_{D}=\frac{1}{2\phi }C_{D}\lambda_{f}U_{c}^{2},$$ (3)

where *λ*_*f*_ (which is non-dimensional) is the total benthos frontal area per unit reef surface area. Below we show that *λ*_*f*_ is a critically-important determinant of the in-reef flow. While evaluation of *C*_*D*_ for a complex coral reef colony is not straightforward, measured values tend to lie in the range 0.5–2 [35–39]. Rather than try to quantify small changes in drag coefficient with coral and flow characteristics, we instead assume a constant value of *C*_*D*_ = 1 for all coral morphologies. This assumption is borne out of practical necessity, as utilization of the framework presented here requires assumption of a coral drag coefficient value (which is otherwise not easily predicted). Later we discuss the implications of our choice and the need for a practical strategy to define *C*_*D*_, particularly for ‘non-front-facing’ morphologies.

For the case where the current is driven by a surface slope (e.g., tidal currents, wave breaking induced currents, etc.), conservation of momentum over the entire depth (*H*) and for a given water surface slope (*S*) requires a balance of the driving hydraulic gradient (left hand side of Eq 4) and the reef benthos drag (Eq 3, right hand side of Eq 4):

$$gHS=\frac{1}{2\phi }C_{D}\lambda_{f}U_{c}^{2},$$ (4)

which can then be rearranged as:

$$U_{c}={\left(\frac{2\phi gHS}{C_{D}\lambda_{f}}\right)}^{\frac{1}{2}}.$$ (5)

The shear stress at the top of the reef benthos (*τ*_*c*_) can also be deduced from a momentum balance, such that the shear velocity at the top of the benthos (u_*_) is given by:

$$u_{*}\equiv \sqrt{\frac{\tau_{c}}{\rho }}{=(g(H-h)S)}^{1/2},$$ (6)

where *g* is the gravitational acceleration constant and *h* is the average height of the reef benthos. In flows over rough colonies, the shear velocity increases linearly with the velocity difference across the benthos (Δ*U*):

$$u_{*}\approx k\Delta U,$$ (7)

where *k* is a coefficient of proportionality [40]. Substitution of (5), (6) and (7) into (1) yields:

$$\beta =1+\frac{u_{*}}{{kU}_{c}}=1+\frac{{\left(g(H-h)S\right)}^{1/2}}{{k(\frac{2\phi gHS}{C_{D}\lambda_{f}})}^{1/2}}=1+k^{*}{\left(\frac{C_{D}\lambda_{f}}{\phi }(1-\frac{h}{H})\right)}^{1/2}$$ (8)

with $k^{*}(=1/\sqrt{2}k)$ a modified coefficient. Eq 8 implies that flow attenuation within a reef benthos tends to zero (i.e. *β* → 1) in the limits of both vanishing benthos frontal area (*λ*_*f*_→0) and emergence of the benthos from the water (*h*/*H* → 1). Later in the discussion we propose how this framework can be applied in practice.

### Numerical model

To demonstrate that the framework in Eq 8 is applicable across a range of coral morphologies, we conducted numerical simulations of flow through colonies of the three coral archetypes (Fig 2). Values of β were estimated across a wide range of coral cover (the ratio of the total coral colony planar area to the total reef surface area, Fig 2), flow depth and speed, and colony orientations relative to the flow (Table 1).

**Table 1:** The range of parameter values considered in the numerical simulations for each archetype coral colony morphology.

Morphology Form	Table	Massive	Branching
**Example Species**	*Acropora hyacinthus*	*Porites lobata*	*Pocillopora edyouxi*
**Coral cover (%)**	0.10, 0.15, 0.20, 0.25, 0.30, 0.40	0.05, 0.10, 0.15, 0.20, 0.25, 0.30, 0.40, 0.50	0.10, 0.15, 0.20, 0.25, 0.30, 0.40
Depth *H* (m)	0.16	0.06, 0.10, 0.16, 0.20, 0.30, 0.40, 0.50	0.16^†^
**Flow direction (°)**	0	0^†^, 45, 90, 180	0
Depth-averaged velocity [m s^-1^]	0.16	0.16^†^	0.05, 0.16, 0.50

^†^Default values for simulations

High-resolution computational fluid dynamics (CFD) simulations were conducted to obtain detailed information about the vertical and horizontal structure of the 3D in-reef flow across a wide range of system variables (Table 1) and coral morphologies (Table 2). We maintained the same coral morphology dimensions in the numerical model that were used in the laboratory validation. Large Eddy Simulations (LES) were run in the CFD solver OpenFOAM [41], which has proven a reliable tool for modelling complex flow around bluff bodies [42] and through aquatic canopies [43], including coral reef benthos [44]. A dynamic kinetic energy subgrid-scale model was employed [as in 45].

**Table 2:** Geometry of each archetypal coral colony morphology used in the numerical models and experimental validation.

Morphology Form	Table	Massive	Branching
**Example Species**	*Acropora hyacinthus*	*Porites lobata*	*Pocillopora edyouxi*
Height, *h* (cm)	3.4	5.1	5.6
Plan area, *A*_*p*_ (cm^2^)	76.7	50.7	63.6
Frontal area, *A*_*f*_ (cm^2^)	17.1	θ = 0° 29.9	95.1
		θ = 45° 33.6	
		θ = 90° 38.1	
		θ = 180° 29.6	

*A*_*f*_ is not simply the total coral area projected into the flow; rather, it is the sum of the projected areas of all coral surfaces (as all coral surfaces will exert drag and create flow attenuation).

*A*_*p*_ was calculated as the coral area projected onto a horizontal plane (i.e. as seen from above).

One coral colony was placed in the horizontal center of the computational domain (as shown in Fig 4B). Cyclic boundary conditions were implemented on all four vertical domain walls, such that the model represents an infinite, uniformly-arranged array of each morphology [46–48]. Coral cover was adjusted by varying the horizontal domain size. While this approach to modelling an irregular natural reef system may appear simplified, we note that, at reef-scale, the drag imposed by regular and random arrays with the same cover is, to first order, equivalent. This has been demonstrated for cylinder arrays, for which the average drag is approximately the same for regular and random arrays (noting that at the individual element level, the drag can indeed vary) [43]. Thus, given that our aim is to computationally resolve the *horizontally-averaged* in-reef velocity, we believe that this simplification is both practically necessary and appropriate.

The numerical domain was generated using utilities in the OpenFOAM package. The blockMesh utility was used to generate the grid, which was then adjusted via the snappyHexMesh tool to fit the mesh to the surface of the coral morphology. Coral surfaces and the underlying bed were specified as no-slip boundaries, with the upper surface of the domain (i.e. the water surface) treated as a non-zero-velocity, zero-flux slip boundary. Four mesh sizes were tested to determine the point of mesh insensitivity (defined as the mesh size where velocity values changed by less than 3%).

All simulations were initially run for 60 s to ensure the flow reached quasi‐steady state, determined from time series of the horizontally averaged velocity at *z* = *h*/2. After the initial spin up, each simulation was continued for a minimum of 10 flow-through periods, during which the velocity data were obtained. While LES provides time-dependent flow output, only the steady-state velocities are presented here. Critically, model results were validated against data from laboratory flume experiments.

### Simulation cases and data analysis

We conducted 21 simulations across the three archetype coral colony morphologies with variable coral cover (5–50%, Table 1). For these simulations, default values of the depth-averaged velocity (0.16 m s^-1^) and water depth (0.16 m) were maintained. To determine the impact of additional system variables on flow attenuation, we maintained a coral cover of 20% and varied (i) the water depth (0.06–0.50 m) for a massive morphology, (ii) the angle of the flow (0-180°) relative to the orientation for a massive morphology, and (iii) the depth-averaged inflow velocity (0.05–0.50 m s^-1^) for the branched morphology. Given that our framework is physically-based, a computationally expensive factorial study was not required here. Rather, it was more important that the key variables controlling the in-reef flow were firstly identified, and then varied over realistic field ranges.

**Fig 3:**
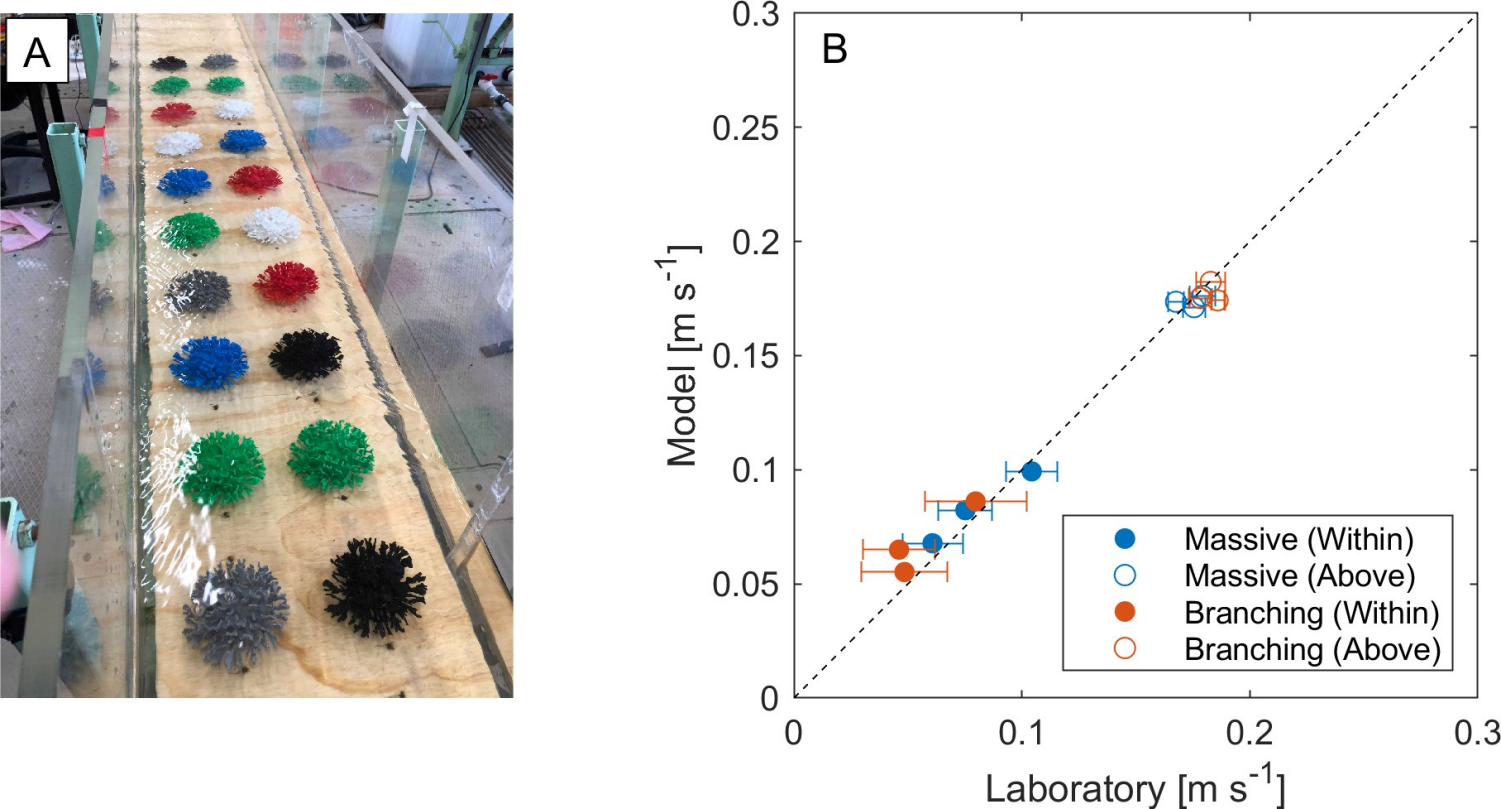
(A) Example of an experimental setup of the branching morphology used for directly measuring velocities in reefs comprised of 3-D printed coral specimens. (B) Comparison of within- and above-reef velocities predicted by the model with those observed in the laboratory. There is excellent agreement between model and experimental velocity values (RMSE = 0.008).

To validate the numerical model, laboratory experiments were conducted in a small (30 cm x 30 cm) open-channel flow flume (Fig 3A). Experiments employed the massive and branched morphologies 3D printed at the same scale as in the numerical model (Table 2). The morphologies were arranged in the flume to form a 2.0-m-long reef flat of specific coral cover (10%, 20% and 30%). Flow velocities above and within the artificial reef benthos were measured at 25 Hz using an Acoustic Doppler Velocimeter located 1.6–2.0 m downstream of the front of the reef. To obtain accurate spatially-averaged in-reef and near-surface velocities, the mean velocity was measured at 6–10 points in horizontal planes both within and above each reef. Representative velocities (*U*_*c*_ and *U*_*∞*_) were taken as depth-averaged values of horizontally-averaged velocities in a 3-cm-tall profile: from *z* = 0.5–3.5 cm (spanning 0.1 ≲ *z/h* ≲ 1) for the in-reef velocity and from *z* = 7.0–10.0 cm (spanning 1.3 ≲ *z/h* ≲ 3) for the near-surface velocity. This provided mean in-reef and above reef velocities with standard errors that were, on average, 10% and 1% respectively. The measured spatially-averaged flow velocities above and within the coral benthos agreed with model results to within an average of 4% (Fig 3B). This demonstrates that the use of a single coral in numerical simulations (with cyclic boundary conditions) generates high-fidelity estimates of the in-reef velocity, and of the velocity ratio *β*.

## Results

### Spatial variation of velocity in reef flows

**Fig 4:**
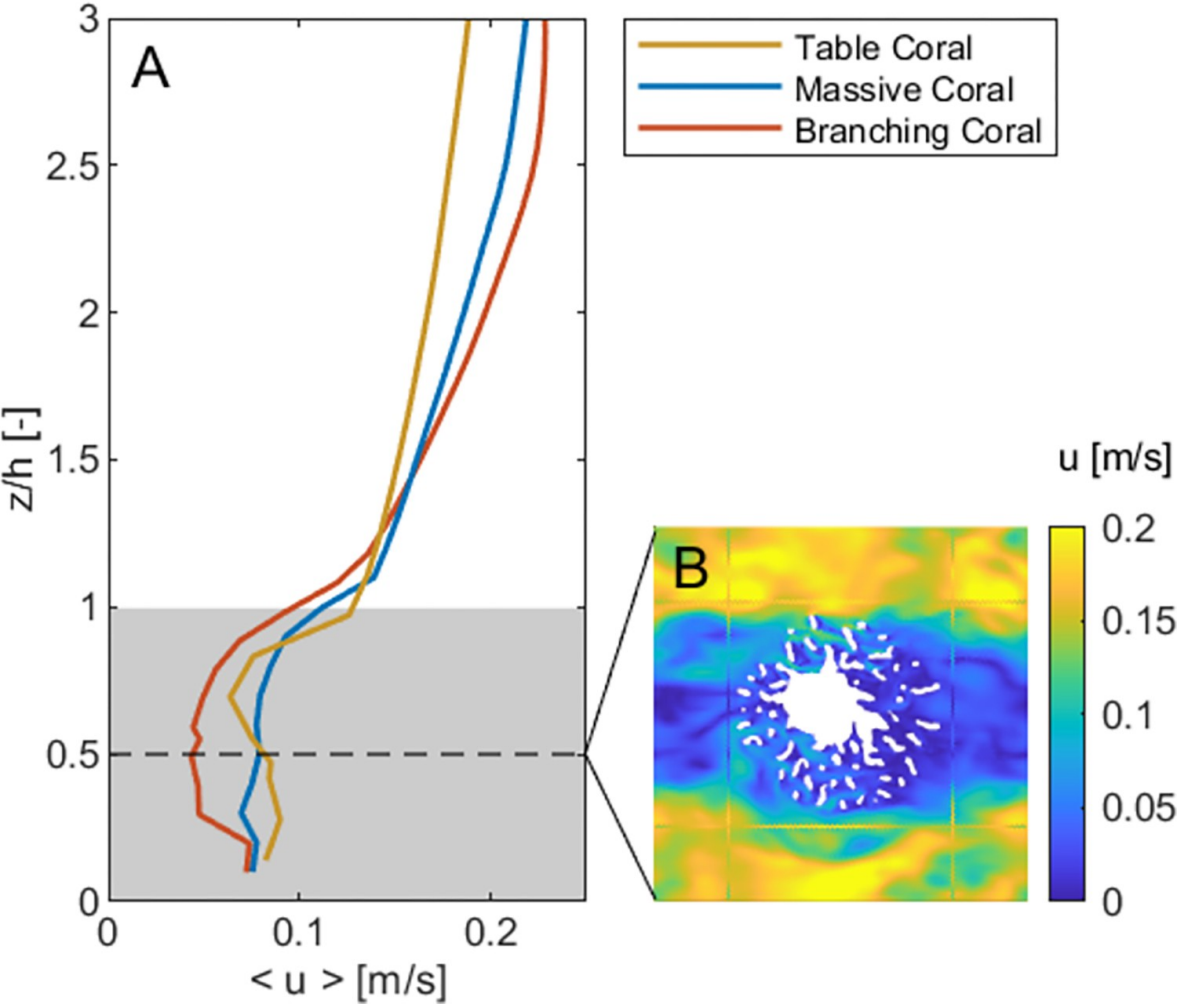
(A) Vertical profiles of the temporally- and horizontally-averaged velocity (*<u>*) for the archetypal coral forms. The shading represents the vertical region used to calculate the depth-averaged in-reef velocity (*U*_*c*_). (B) The significant horizontal variability (at *z/h* = 0.5) of the instantaneous flow field within a reef consisting of the branched morphology.

Vertical profiles of the temporally- and horizontally-averaged velocity (<*u>(z)*) are shown in Fig 4A for all three colony types with a coral cover of 20%. In all cases, there is a region of roughly uniform, attenuated flow that extends from the seabed to the top of the coral colonies (i.e. 0 < *z*/*h* < 1), and a uniform high-velocity region well above the colonies. These regions are separated by a strong shear layer [24] where the flow velocity changes rapidly. In the numerical model, we evaluated the near-surface velocity (*U*_*∞*_) as that 0.01 m below the water surface, and *U*_*c*_ as the mean velocity in the region 0 < *z* < *h* across the entire horizontal extent of the domain (i.e. a horizontally- and vertically-averaged velocity, Fig 4A). While the choice of how *U*_*∞*_ and *U*_*c*_ are evaluated non-trivially impacts the value of *β*, it does not alter the validation of the framework and the predictive capacity developed in this paper.

There is also substantial horizontal variability of the in-reef flow velocity (Fig 4B). For the branched colony case in Fig 4, there are distinct regions of low flow within its branches, as well as within its wake, with faster channelized flow in the gaps between adjacent colonies. Far above the branching colony the flow velocity is 0.20 m/s, while at mid-height (*z*/*h* = 0.5) the velocity is 0.048 ± 0.055 m/s (horizontal mean ± spatial standard deviation, Fig 4B).

### Impact of key system parameters on flow attenuation

**Fig 5:**
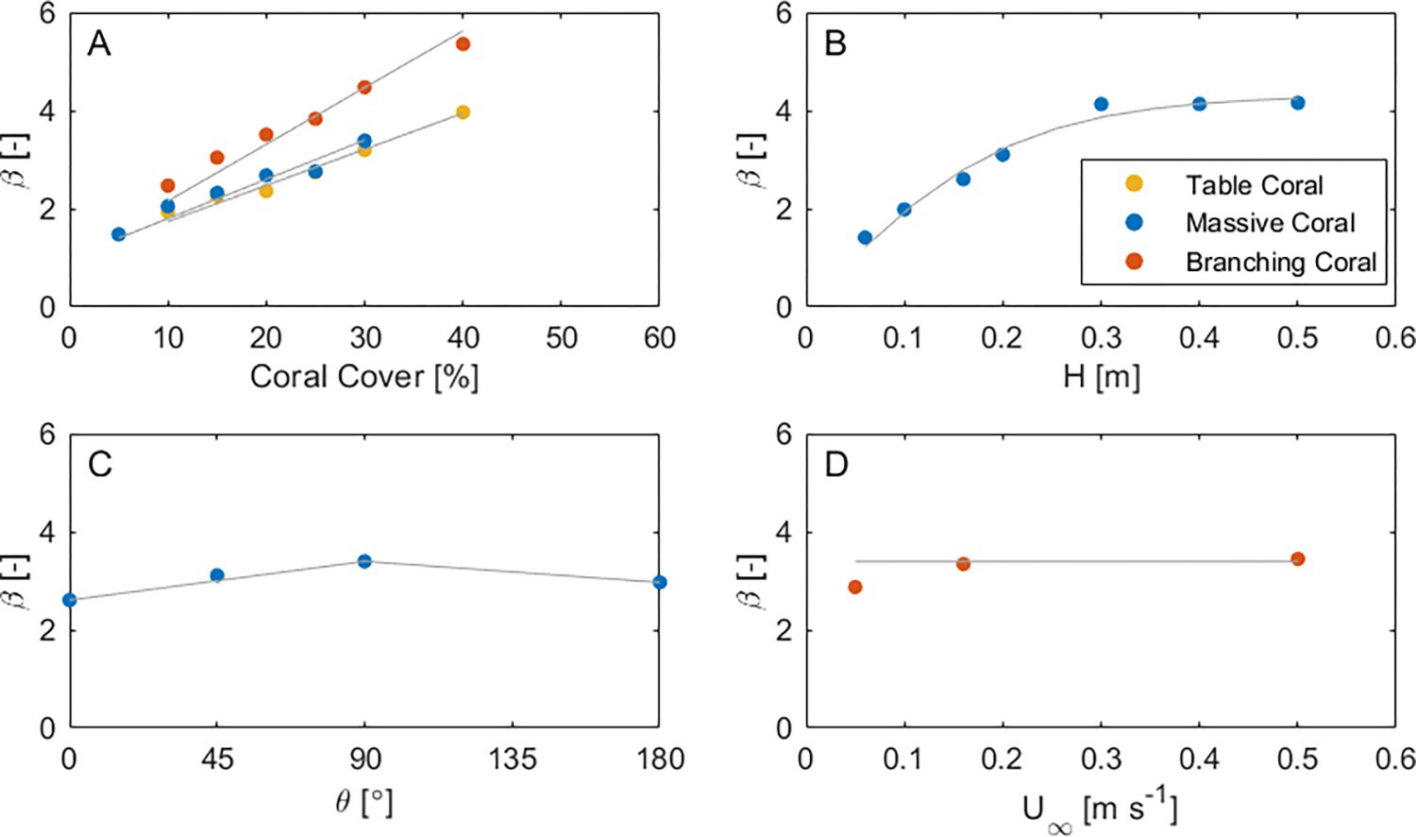
The extent of flow attenuation *β* for the three archetypal coral forms is impacted by: (A) the colony coral cover, (B) the water depth *H*, (C) the angle of the approaching flow to the morphology *θ*, and (D) the near-surface velocity *U*_*∞*_. Where particular parameters were not varied, they took the default values in Table 1: coral cover of 20%, a depth of 0.16 m, a depth-averaged velocity of 0.16 m s^-1^ and *θ* = 0°. The coral cover and water depth have the greatest influence on *β*. The grey lines demonstrate general trends, but do not represent a particular functional form.

A wide range of *β* was observed in this study (1.4–5.3, Fig 5) across a relevant range of key system variables (coral cover, coral morphology, water depth, coral orientation relative to the flow and flow velocity). Here, we demonstrate the extent of flow attenuation, and its sensitivity to coral morphology and other system parameters.

#### Coral cover

For all morphologies, there is a linear increase of *β* (from 1) with increasing coral cover (Fig 5A). For a given water depth, coral cover is the parameter with the greatest influence on *β*, with the greatest values for cover exceeding 30%.

#### Morphology

For given coral cover, the branching form consistently generates the greatest flow attenuation (Fig 5A). This is due to all the individual branches contributing to a significantly enhanced total frontal area per unit reef surface area (Table 2). That is, for a fixed amount of biomass, corals that have thin, branching entities (that maximizes the organismal frontal area) will impact flow velocity much more so than a near-spherical shaped form (a shape which may minimize organismal frontal area).

#### Water depth

Consistent with Eq 8, *β* increases with the water depth in shallow flow before becoming independent of water depth in deeper flow (here when *H* ≥ 0.3 m, or *H/h >* 6, Fig 5B).

#### Orientation

*β* is moderately sensitive to the orientation of the coral morphology relative to the flow for the massive morphology (Fig 5C), as the frontal area projected into the flow changes (and thus *λ*_*f*_ in Eq 8). Here, an orientation of 0° indicates that the narrow end of the massive morphology is oriented into the flow (see Fig 1). For a 90° change in the flow direction, there is a 27% increase in the frontal area projected by the morphology, and a corresponding 30% increase in *β* (Fig 5C).

#### Flow velocity

There is negligible variation of *β* with the depth-averaged velocity (Fig 5D). This implies that the model Reynolds numbers (Re = *Ud*/*ν*, where *ν* is fluid kinematic viscosity and *d* is a characteristic width), which are indicative of field values, are sufficiently high that *β* is independent of Reynolds number (and thus velocity itself).

### Predictive capacity of analytical framework

Assuming an invariant drag coefficient, the analytical framework presented in Eq 8 predicts that (*β*─1) will increase with α^1/2^, where

$$\alpha =\frac{\lambda_{f}}{\phi }\left(1-\frac{h}{H}\right).$$ (9)

**Fig 6:**
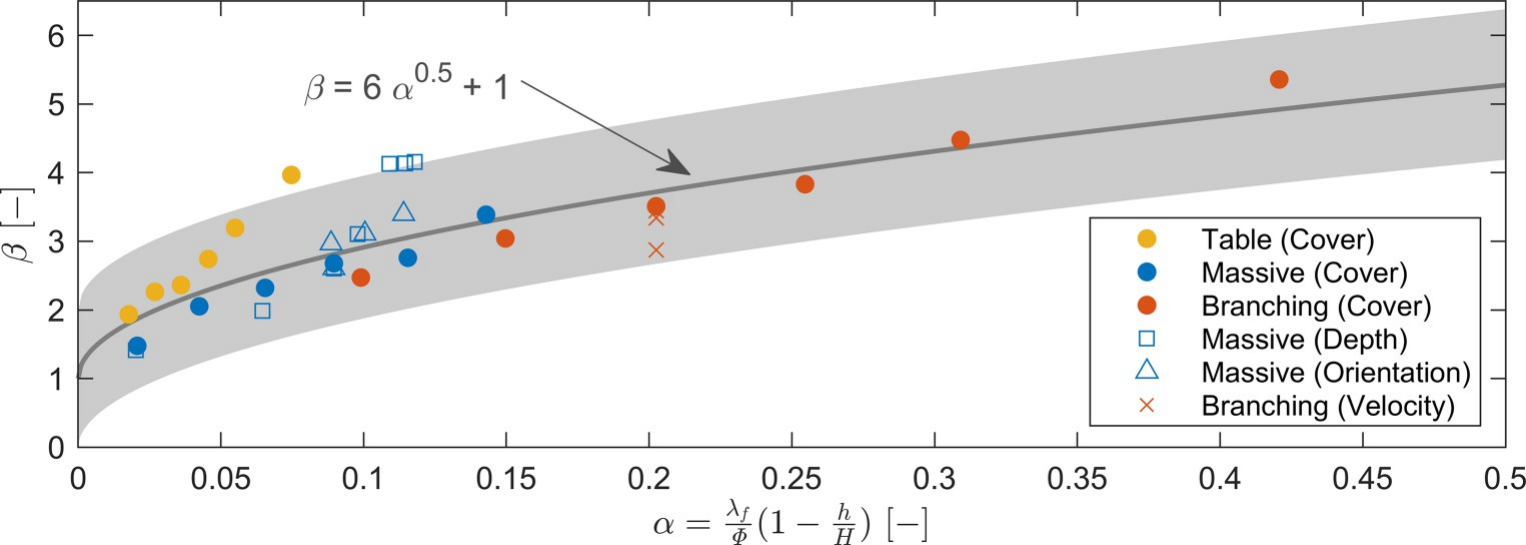
The performance of the predictive model (Eq 8) in collapsing the effects of reef colony morphology, coral cover, water depth, orientation and flow velocity into a single predictor variable for in-reef flow attenuation. The solid markers indicate the coral cover cases for the three archetypal coral forms. The open markers indicate cases where depth, orientation or velocity (as indicated in brackets in the legend) were varied systematically for one coral morphology. The equation for the line of best fit is indicated on the plot, with the shading representing the 95% prediction interval.

Indeed, across all the numerical simulations conducted here and across all the dimensions of variation considered in Fig 5 (coral morphology, coral cover, water depth, orientation angle and flow velocity), there is an excellent agreement between the data and the prediction curved for *β* and *α* (*R*^*2*^ = 0.70, Fig 6). The measure of this agreement increases to *R*^*2*^ = 0.86 when the three noticeably different table cases are excluded (we discuss the reason for this in the next section). The line of best fit in this figure represents the relationship *β*─1 ≈ *α*^1/2^. The agreement of the data across morphologies as well as the agreement of the functional forms of the line of best fit and Eq 8 validate the analytical framework presented here (which has a single predictor variable, α).

## Discussion

### Predictability of flow on coral reefs

The agreement of the data with the curve of *β* on *α* (Fig 6) is a fundamentally-important result, as it implies that the canopy-averaged in-reef flow attenuation within the benthos of a coral reef can be predicted through knowledge of three core parameters regardless of coral species: the benthos frontal area per unit reef area, the height of the benthos and the water depth. Deviations from the predicted curve (Eq 8) are likely to be associated with changes in the true values of coral drag coefficient *C*_*D*_, which can be reasonably expected to vary according to morphology but were assumed constant in our estimation of α. For example, the numerical experiments show slightly greater flow attenuation for the table colony with high coral cover (Fig 6). This deviation can be explained by a unique feature of this morphology: namely, the large plan area (rather than frontal area) of these colonies. This geometry has the effect of providing flow resistance in the form of skin friction that is not embedded within the analytical framework (and ultimately serves to increase the effective value of the drag coefficient in Eq 3). Consistent with this unaccounted-for flow resistance, the flow attenuation of the table colony is *higher* than would otherwise be expected. A better understanding of the variation of *C*_*D*_ with coral morphology would assist to constrain these deviations and we suggest that initial efforts could focus on accounting for non-front-facing morphologies (e.g., table corals). While *C*_*D*_ is expected to vary based on the flow and morphology, this variation is somewhat small relative to the natural variation of other components that make up the predictor variable (*α*). Furthermore, the scale at which inhomogeneity of coral morphologies affects drag warrants further investigation. While studies of cylinder arrays suggest that the average drag is similar for both homogeneous and inhomogeneous arrays, at an individual colony scale the drag can be expected to be different [43]. Notwithstanding these areas that required further investigation, to first-order we suggest that the general model (Fig 6) is appropriate for estimating in-reef velocities across the full range of morphologies.

### Implementation of framework in practice

**Fig 7:**
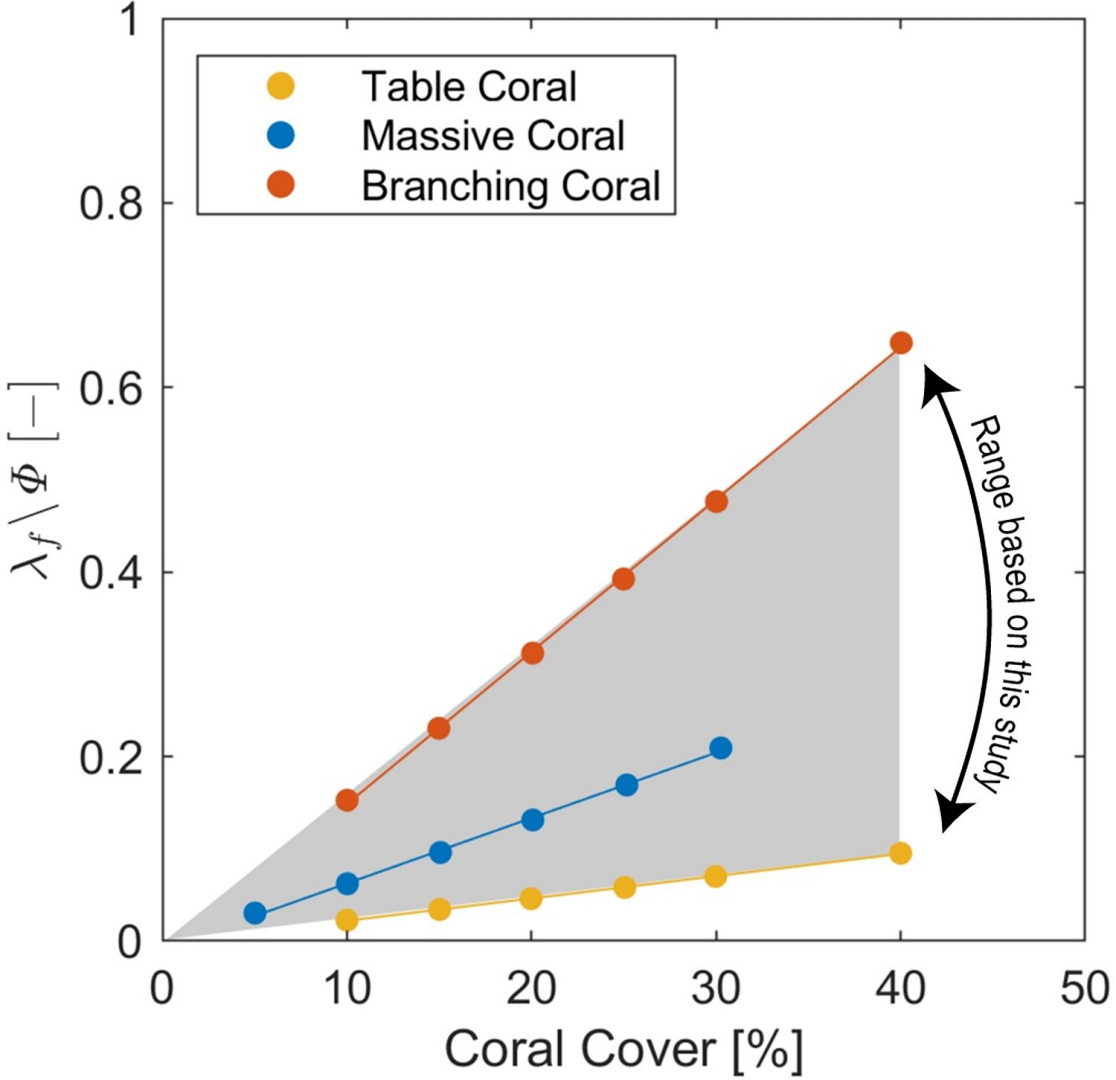
The allometric relationship between coral cover and the resultant value of *λ*_*f*_/*ϕ* for three archetypal benthos colony forms (“table”, “massive” and “branching”) which are based on observations of the representative species Acropora hyacinthus [15], Porites lobata [15] and Pocillopora edyouxi [16], respectively. The markers indicate data from the numerical simulations. The shaded area roughly represents the range of typical values based on the results of this study.

To apply this framework, four key data are required: (1) a spatial map of reef benthos species (to ultimately determine reef frontal area and porosity), (2) the height (*h*) of the reef benthos, (3) the total water depth (*H*) and (4) the surface velocity (*U*_*∞*_). We recognize that not all of this data will always be available, however we believe that it can be practically obtained. Spatial maps of reef benthos are routinely assembled by diver [49, 50], drone [51], aerial [52] or satellite [53, 54] methods. These maps of coral cover and occasionally species composition, which are more important than the orientation of the reef benthos to the flow, can be used to inform the choice of *λ*_*f*_/*ϕ* (the component of α that is determined by coral cover and morphology). This requires quantitative allometric relationships between *λ*_*f*_/*ϕ* and the more easily-obtained measure of coral cover. For the three archetypes employed here, indicative forms of such relationships are presented in Fig 7. We believe that these curves represent the typical range of forms of the *λ*_*f*_/*ϕ*-coral cover relationship, a table coral minimizes *frontal* area per unit biomass while branches serve to maximize it. Such curves can be developed for other morphological forms with relative ease using techniques such as CT scanning [55] of colony fossils.

The three remaining pieces of data are less difficult to determine. The geometric height of the benthos can be obtained in a number of different ways including from representative spot measurements or vessel mounted echo-sounders [56], as well as from aerial LIDAR [31], Structure from Motion imagery [51, 57, 58] or even from satellite imagery [59]. The water depth can be determined from surface elevation data or *in situ* hydrodynamic measurements with wave or tide gauges [60]. Finally, the near-surface velocity can be obtained by surface tracking of objects such as drifters [61] or by tilt meters and acoustic instruments [1, 51, 62] that can measure velocity well above, but not within, the reef. All methods are commonly used in hydrodynamic and monitoring studies.

An obvious consideration is how this framework can be applied to a reef composed of a range of coral species. The nature of this framework is such that mixed benthos can be characterized as the sum of the frontal areas of its constituent parts, contributing to a ‘total’ value of *λ*_*f*_/*ϕ* in Fig 6. Importantly, the canopy frontal area per unit reef surface area controls the resistance exerted on the flow through form drag. Practically, this area can be estimated spatial maps of reef benthos and allometric relationships of the form shown in Fig 7.

### Implications for coral reef process understanding, resilience and restoration

Traditional approaches used to describe the hydrodynamic impact of coral reef benthos is through a friction coefficient that describes the impact of reef on the overlying flow [35]. This approach, however, does not allow prediction of the flow within the benthos, which is relevant to the wide range of reef processes that govern coral reef health and function. The framework proposed in this study enables this flow to be estimated through the inclusion of Eq 8 within established numerical models (for example as a wall-type function) and, importantly, without the need to explicitly resolve the physical structure of the benthos in these models at regional scales. This is a major step forward in understanding the range of environmental transport processes on reef systems (of, for example, heat, nutrients and in some cases biota), the future of reef-adjacent coasts (i.e., the supply and loss of sediment) and quantitative design of reef restoration activities.
